# tsAMP: a strain-level antimicrobial peptide identification framework based on large language models and pathogen genomic variation

**DOI:** 10.3389/fmicb.2026.1842380

**Published:** 2026-07-02

**Authors:** Haimeng Li, Han Gao, Jian Tian, Xin Wang, Rui Jiang, Ting Chen, Hui Chen, Yuqing Yang, Congmin Zhu

**Affiliations:** 1 State Key Laboratory of Networking and Switching Technology, Beijing University of Posts and Telecommunications, Beijing, China; 2 State Key Laboratory of Animal Nutrition and Feeding, Institute of Animal Sciences, Chinese Academy of Agricultural Sciences, Beijing, China; 3 Department of Ultrasound, Peking Union Medical College Hospital, Beijing, China; 4 Chinese Academy of Medical Sciences, Peking Union Medical College, Beijing, China; 5 Bioinformatics Division and Center for Synthetic and Systems Biology, Department of Automation, Beijing National Research Center for Information Science and Technology, Tsinghua University, Beijing, China; 6 Institute for Artificial Intelligence and Department of Computer Science and Technology, Tsinghua University, Beijing, China; 7 School of Biomedical Engineering, Capital Medical University, Beijing, China; 8 Beijing Key Laboratory of Fundamental Research on Biomechanics in Clinical Application, Capital Medical University, Beijing, China

**Keywords:** antibacterial capability, antimicrobial peptides, large language model, minimum inhibitory concentration, pathogens

## Abstract

**Introduction:**

Facing the global threat of multidrug-resistant bacteria, antimicrobial peptides (AMPs) represent a promising alternative to conventional antibiotics.

**Methods:**

To improve computational AMP identification and accuracy of strain-level MIC prediction, we developed tsAMP, a comprehensive framework integrating the ESM-1v protein language model with multidimensional feature extraction. The model was trained on AMP and metagenome-derived non-AMP sequences.

**Results:**

tsAMP achieved an F1-score of 0.958 for AMP identification, outperforming state-of-the-art tools. For bacterial inhibition prediction, tsAMP consistently maintained F1-scores above 0.8 across 33 pathogenic species. In strain-specific MIC prediction, it attained high performance (MSE = 0.214, *R*^2^ = 0.634) for 10 bacterial species’ strains. To assess predictive reliability, the model was benchmarked against published experimentally determined MIC values for AMPs targeting *Micrococcus luteus*, yielding low prediction error (MSE = 0.1489) and strong ranking consistency (NDCG = 0.791). Computational benchmarking against published relative MIC data for diverse *E. coli* strains further demonstrated the model’s ranking accuracy (NDCG > 0.85) and consistent strain-level differentiation. Applied to the Mgnify_genome database, tsAMP identified 8,277 putative AMP candidates in *silico* and revealed distinct predicted antimicrobial activity patterns across pathogens.

**Discussion:**

tsAMP provides a computational framework to facilitate the identification of AMP candidates and support prioritization for downstream experimental characterization. The code is available on GitHub at https://github.com/YangLab-BUPT/tsAMP.

## 1 Introduction

The emergence of multidrug-resistant bacteria and the so-called “superbugs” poses a serious threat to global public health and underscores the urgent demand for novel antimicrobial agents (Cook and Wright, 2022; D’Costa et al., 2011). Although the discovery of antibiotics revolutionized the treatment of bacterial infections and their subsequent widespread application in medicine, agriculture, and food production has provided enormous benefits, their misuse and overuse have raised serious concerns regarding antibiotic resistance (Ma et al., 2024; Ventola, 2015). In this context, antimicrobial peptides (AMPs) have attracted considerable attention as potential alternative therapeutic agents (Hancock and Sahl, 2006). Bacterial resistance to AMPs has been extensively documented through multiple mechanisms, including membrane remodeling, proteolytic degradation, active efflux systems, and biofilm-associated protection (Joo et al., 2016). AMPs are small, naturally occurring molecules, typically composed of fewer than 100 amino acids, and they play a vital role in the innate immune system (Bahar and Ren, 2013). These peptides possess a cationic charge (usually between +2 to +9) and an amphipathic structure that includes hydrophobic and hydrophilic domains (Farkas et al., 2017; Moravej et al., 2018). These structural properties, such as charge, hydrophobicity, and conformation, endow AMPs with a broad-spectrum antimicrobial activity and immunomodulatory, wound-healing, and anti-tumor functions, which make them attractive candidates for novel therapeutic strategies (Bahar and Ren, 2013; de la Fuente-Núñez et al., 2017).

Computational approaches have been widely applied to AMP identification. In machine learning-based studies, Hosein Khabaz proposed a hierarchical approach that simultaneously performs binary classification (AMP vs. non-AMP) and predicts activity against *Staphylococcus aureus* (Khabaz et al., 2023). CalcAMP introduces a predictive framework specifically tailored for short peptides (<35 amino acids), which leverages publicly available data to assess antimicrobial activity against Gram-negative and Gram-positive bacteria (Bournez et al., 2023). AGRAMP utilizes a random forest (RF) model combined with N-gram peptide sequence representations using 3- and 9-letter reduced amino acid alphabets, successfully capturing key sequence patterns and functional motifs associated with AMP activity (Shao et al., 2024). Despite these advances, conventional machine learning methods remain limited in their ability to model complex sequence patterns and nonlinear relationships (Jordan and Mitchell, 2015).

Deep learning has significantly advanced AMP prediction research (Liu et al., 2024). Initial studies employed relatively simple architectures: for instance, iAMPCN utilizes convolutional neural networks (CNNs) for the multilabel prediction across 22 functional categories, while AMPlify optimizes sequence feature extraction through the introduction of attention mechanisms (Li et al., 2023; Xu et al., 2023). With technological progress, more sophisticated architectures have emerged. AMP-BERT incorporates transformer models-originally developed for natural language processing-for improved interpretability via attention-based visualization of key residues (Lee et al., 2023). Pang *et al.* further enhanced multi-functional prediction by integrating transformers with asymmetric loss functions (Pang et al., 2022). FC Fernandes pioneered the application of geometric deep learning to AMP functional prediction and effectively addressed the challenge of modeling non-Euclidean structural features that traditional methods fail to capture (Fernandes et al., 2023).

Moreover, models such as AI4AMP, integrate the latest AMP datasets with rigorously unbiased negative samples and employ hybrid CNN-long short-term memory (LSTM) architectures; achieving a prediction accuracy exceeding 90% (Lin et al., 2021). APEX and esAMPMIC demonstrate strong performance in minimum inhibitory concentration (MIC) prediction by employing ensemble learning frameworks that integrate CNN and Bi-LSTM models (Chung et al., 2024; Wan et al., 2024). More recently, Diff-AMP innovatively combines diffusion models with transfer learning to achieve fully automated AMP design, spanning sequence generation to optimization (Wang et al., 2024). Collectively, these advances have considerably improved the predictive performance and interpretability of AMP models and offer new avenues to address antimicrobial resistance.

Nevertheless, current approaches still face key challenges. Many models rely on a single feature dimension without integrating the multidimensional attributes of AMPs. For example, AI4AMP considers only the physicochemical properties of individual amino acids, without capturing sequence-level information or the overall physicochemical characteristics of AMPs. Similarly, AMPlify and AMP-BERT focus only on sequence-level feature extraction. Another critical limitation arises from negative-sample selection in binary classification tasks. Excessive disparity between the selected negative and positive samples may yield favorable test performance, but leads to poor generalization capability and high false-positive (FP) rates in practical applications. Furthermore, most existing methods cannot predict AMP MIC at the strain level. The same AMP may exhibit markedly varied MIC values against distinct strains of the same bacterial species. For example, cathelicidin AM (1–21) demonstrated an MIC value of 21 μg/mL against *Escherichia coli* ATCC 4157, but its MIC increased to 140 μg/mL against *Escherichia coli* ATCC 51659 (Pirtskhalava et al., 2021). This variability was further validated by the BroadAMP-GPT model: AMP_s14 showed a potent inhibitory activity against *Enterococcus faecium* QF31, and its MIC exceeded 128 μg/mL for strain SF23-1 (Li et al., 2025). Although some approaches such as APEX and esAMPMIC enable species-level predictions, they remain restricted in two key aspects: they can only analyze fixed pathogenic species and cannot capture strain-level variations within the same pathogenic species, which leaves substantial room for improvement.

To overcome these limitations, we propose tsAMP (strain-level Antimicrobial Peptide identification framework), a novel comprehensive model that integrates the ESM-1v protein large language model (LLM) with multidimensional feature extraction for both general AMP prediction and strain-level MIC estimation. By combining AMP sequence-based and physical–chemical property multidimensional features, tsAMP achieves a generic framework for AMP identification. During training, the problem of sample imbalance and excessive difference between the training samples, which leads to poor model generalization capability, was considered to reduce the FPs of AMP prediction. tsAMP can not only identify AMP sequences but also accurately predict their MIC values against diverse pathogens, including different strains of the same species, making it a computational tool for AMP candidate identification and prioritization for experimental characterization.

## 2 Materials and methods

### 2.1 Datasets merging and preprocessing

The extensive resources of natural antimicrobial peptides curated by established databases such as the APD have provided a comprehensive framework for classification and characterization, significantly catalyzing the application of deep learning in the discovery of novel AMPs (Wang, 2023). The initial AMP positive dataset was compiled from two major databases: dbAMP2.0 (Jhong et al., 2022) and DBAASP (Pirtskhalava et al., 2016), both based on the founding database APD (Wang and Wang, 2004). After removing duplicate sequences, a final curated collection of 19,412 unique AMP entries was obtained. MIC values reported in μg/mL in dbAMP2.0 and DBAASP were converted to μM by dividing the peptide molecular weight and multiplying by 1,000, with findings standardized to two decimal places, and finally transformed into a log μM scale (Sharma et al., 2023). This conversion followed standard microbiological practice for the effective balance of the distribution of MIC values across orders of magnitude and improving data comparability and statistical robustness. For the detection of AMPs, the test sets were constructed by selecting sequences from the APD3 and LAMP2 databases, from which all redundant entries were removed (Wang et al., 2016; Ye et al., 2020).

The non-AMP dataset was collected from UniProt (UniProt Consortium, 2019) and GMGC (UniProt Consortium, 2019). All reviewed protein sequences from the UniProt, with a sequence length of ≤100 amino acids and an annotation score ≥ 2 were selected as the initial negative-sample dataset. Sequences with annotations related to antimicrobial, antibiotic, amphibian defense, or antiviral protein, were excluded (Zhang et al., 2024). To enhance the diversity of negative samples, immune-related protein sequences (≤100 amino acids) from environmental sources—including animals, plants, and humans—were obtained from UniProt and incorporated into the dataset, and human-related environmental protein sequences were retrieved from GMGC.

Sequences with lengths between 50 and 100 amino acids were screened out from the original dataset. Exactly 50% of these sequences were randomly selected for segmentation processing and evenly divided into two independent sequences to expand the number of short peptides (<50 amino acids). Meanwhile, 10,000 sequences longer than 100 amino acids were randomly selected and fragmented into peptides of 10, 20, 30, 40, and 50 amino acids (Sidorczuk et al., 2022; Veltri et al., 2018). BLAST analysis was then performed to identify sequence similarities between negative sequences and known AMP-positive sequences (Ye et al., 2006), with the sequence identity threshold set to 20%. All duplicate sequences were removed, yielding a final set of 117,811 negative-sample sequences.

### 2.2 Deep-learning-based AMP recognition

The ESM-1v protein language model was employed to extract features from all protein sequences and generate 1,280-dimensional vector representations for the effective capture of the deep semantic information of the sequences. Normalized hydrophobicity, net charge at physiological pH, amphipathicity index, and isoelectric point (pI) were extracted from the peptide sequences. The hydrophobicity value of each amino acid residue was calculated based on the Kyte–Doolittle hydrophobicity scale (Kyte and Doolittle, 1982). Net charge was calculated using Biopython’s ProteinAnalysis module at pH 7.4 (Cock et al., 2009). The amphiphilicity index was calculated through quantifying the difference between the number of hydrophobic residues (alanine, cysteine, and seven others) and hydrophilic residues, and the result was normalized by sequence length to yield a score ranging from 0 to 1. The pI was determined through the Bjellqvist method (Bjellqvist et al., 1982).

A deep learning-based module, tsAMP-I, was built to recognize the AMP sequence. tsAMP-I contains three hidden layers with 256, 128, and 128 units, respectively. The input layer contains 1,284 units representing both sequence features and physicochemical properties of AMPs. Each hidden layer is followed by a ReLU activation function and a dropout layer to prevent overfitting. Model training process involved the binary cross-entropy loss function with Logits (BCEWithLogitsLoss) as the loss function. Model parameters were updated using the Adam optimizer (with the learning rate and weight decay coefficient set to 0.0001 and 0.01, respectively). To improve model robustness, Gaussian noise enhancement was applied to positive samples during training, and environmental sequences were incorporated into the negative dataset.

### 2.3 Species- and strain-level MIC prediction modules

The classification module, tsAMP-C, predicts the approximate range of MIC values for novel peptide sequences during binding to various target species. Trained using AMPs with experimentally validated target species from dbAMP2.0 and DBAASP databases, the ESM-1v protein language model was used to extract 1,280-dimensional feature vectors from both AMP and target species sequences, which were then concatenated into 2,560-dimensional vectors.

The inhibitory potency of AMPs against 33 target species was predicted by establishing classification criteria based on four widely used MIC thresholds (2, 4, 8, and 16 μM). AMPs with MIC values below these thresholds were classified as demonstrating a strong inhibition (class 0), and those above were categorized as showing a weak inhibition (class 1). We addressed data scarcity using a GAN for data augmentation during model training. The generator network consists of three fully connected layers with ReLU activation and progressively transforms the latent input through 256 and 512 hidden units before producing normalized outputs via tanh activation. Meanwhile, the discriminator utilizes a similar three-layer architecture with 512 and 256 hidden units that process input data through ReLU activation before final classification using a sigmoid activation function. Both networks utilize a consistent dropout rate of 0.3 for regularization, with the generator’s tanh output layer ensuring values in the [−1, 1] range and the discriminator’s sigmoid output providing probabilistic classification between real and synthetic samples. The architecture of tsAMP-C consists of two hidden layers (256 and 64 units), and the final implementation includes 33 sub models, each specialized and optimized for one target species.

For the prediction of precise MIC values, the regression module, tsAMP-CS, was developed using target species with strains’ sizes exceeding 1,000 entries in the AMP database. Trained using AMPs with experimentally validated target species from dbAMP2.0 and DBAASP databases, the ESM-1v protein language model was used to extract 1,280-dimensional feature vectors from both AMP and target strains sequences (sequences of the target strains were downloaded from NCBI, extracted features, and then took the mean representations), which were then concatenated into 2,560-dimensional vectors. During training, various strains belonging to the same bacterial species were merged for regression analysis. Multiple data augmentation methods—including Gaussian noise, dropout noise, random scaling, and mixup interpolation—were employed to improve generalization. The model architecture comprises four layers: an input layer (2,560 units), two hidden layers (512 and 128 units), and a single-unit output layer for MIC prediction, with mean squared error (MSE) loss as the optimization objective.

### 2.4 Performance prediction of three tsAMP modules

Precision, recall, F1-score, specificity, MCC, and accuracy were used to evaluate the classification models (tsAMP-I and tsAMP-C) in order to comprehensively assess their reliability in AMP identification, their ability to detect potential AMPs, and their robustness under class imbalance between positive and negative samples:


$$\text{Precision}=\frac{\mathrm{TP}}{\mathrm{TP}+\mathrm{FP}}$$

$$\text{Recall}=\frac{\mathrm{TP}}{\mathrm{TP}+\mathrm{FN}}$$

$$\text{Specificity}=\frac{\mathrm{TN}}{\mathrm{TN}+\mathrm{FP}}$$

$$F1\;\text{score}=2\times \frac{\text{precision}\times \text{recall}}{\text{precision}+\text{recall}}$$

$$\mathrm{MCC}=\frac{\mathrm{TP}\times \mathrm{TN}-\mathrm{FP}\times \mathrm{FN}}{\sqrt{\left(\mathrm{TP}+\mathrm{FP})(\mathrm{TP}+\mathrm{FN})(\mathrm{TN}+\mathrm{FP})(\mathrm{TN}+\mathrm{FN}\right)}}$$

$$\text{Accuracy}=\frac{\mathrm{TP}+\mathrm{TN}}{\mathrm{TP}+\mathrm{FP}+\mathrm{FN}+\mathrm{TN}}$$

In the tsAMP-I module, TP corresponds to AMPs correctly identified, and FN represents AMPs erroneously classified as non-AMPs. For tsAMP-C’s binary classification, AMPs with MIC values below the predefined threshold were designated as the positive class (class 0, strong inhibition), and those above the threshold were designated as the negative class (class 1, weak inhibition). Accordingly, TP denotes cases where both the predicted and experimentally determined MIC values fall below the threshold (i.e., the model correctly identifies a strongly inhibitory AMP), FP denotes cases where the model predicted strong inhibition (MIC < threshold) but the experimental MIC exceeded the threshold, FN denotes cases where the model predicted weak inhibition but the experimental MIC was below the threshold, and TN denotes cases where both predicted and experimental MIC values exceeded the threshold.

For the effectiveness of the regression module (tsAMP-CS), MSE and R^2^ were utilized as prediction metrics, and PCC was used to predict the linear relationship between true and predicted values. A coefficient close to 1 indicates a strong linear trend consistency. To prevent data leakage between training and test sets, all peptide sequences were first clustered using CD-HIT at a 40% sequence identity threshold, and entire clusters were assigned exclusively to either the training or test set, ensuring that no test peptide shares more than 40% identity with any training peptide (Todd et al., 2001; Xu et al., 2023). All performance metrics were calculated using bootstrap resampling with 1,000 iterations to estimate the confidence intervals and standard deviations (DiCiccio and Efron, 1996).

Given the large number of negative samples in tsAMP-I, five independent subsets of negative samples were randomly selected during training, and their averages were computed for performance prediction. For both tsAMP-C and tsAMP-CS, fivefold cross-validation was performed, and the average performance across folds was reported.

## 3 Results

### 3.1 LLM-based AMP identification and classification framework

We developed a novel predictive framework, tsAMP, based on the ESM-1v protein language model, comprising three modules: tsAMP-I, tsAMP-C, and tsAMP-CS (Meier et al., 2021). The tsAMP-I module identifies AMP versus non-AMP sequence using curated AMP and negative datasets that encompass comprehensive AMP-related information, thereby enhancing the generalization capability of AMP prediction. The tsAMP-C and tsAMP-CS modules utilize ESM-1v to extract features from AMPs, bacterial species, and strains. Specifically, tsAMP-C predicts the inhibitory potency of AMPs against 33 bacterial species, and tsAMP-CS calculates the precise MIC values of AMPs against strains from 10 prevalent target species based on strain-specific features. In addition, tsAMP-CS can extend MIC prediction to strains of other pathogenic bacteria.

**Figure 1:**
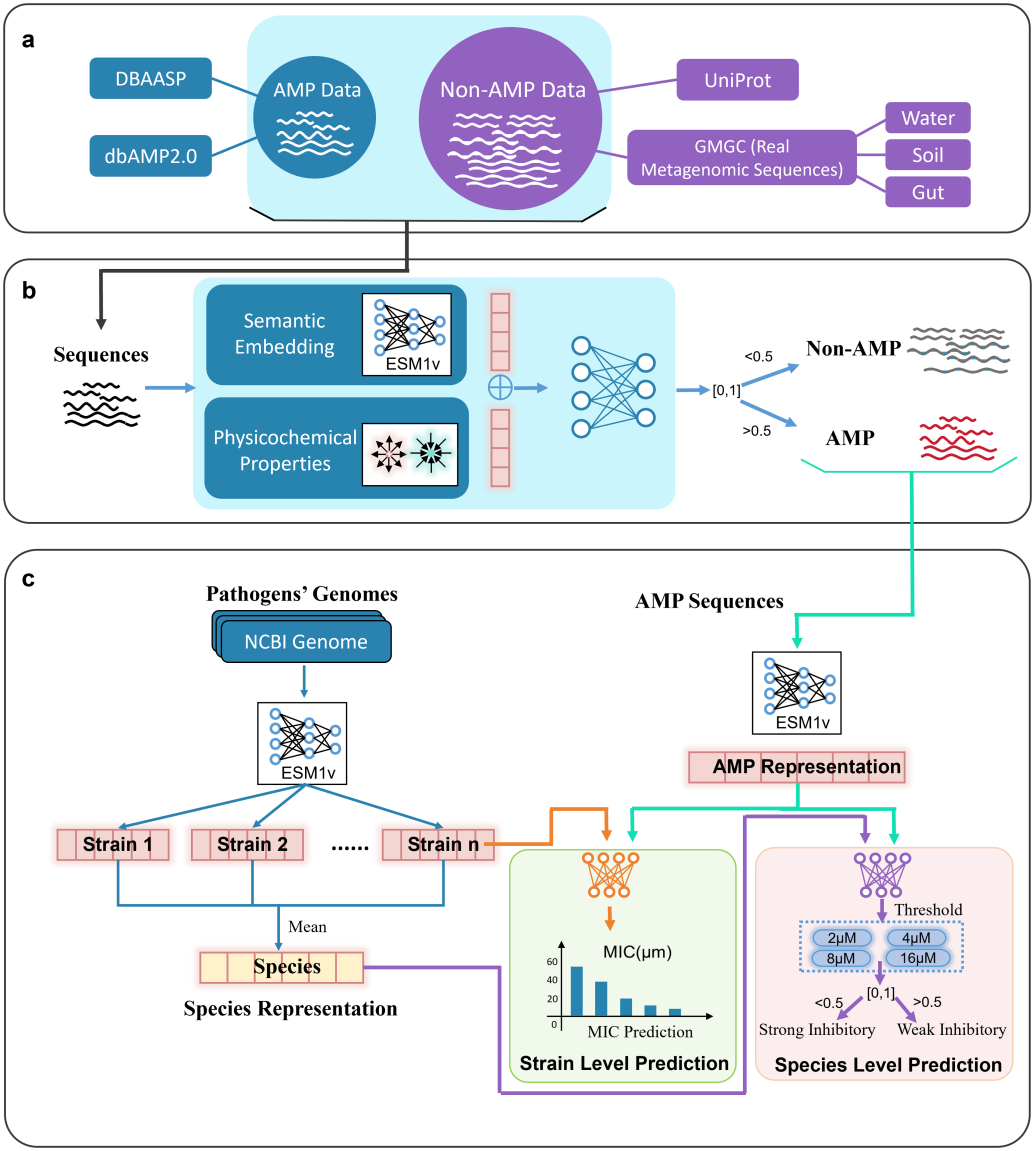
Overview of the tsAMP framework. **(a)** Composition of datasets used in tsAMP. **(b)** The tsAMP-I module for AMP identification. Protein features and physicochemical properties of the sequence were extracted, and tsAMP-I predicts whether the sequence is an AMP. **(c)** The tsAMP-C and tsAMP-CS modules for MIC prediction. tsAMP-C predicts the overall inhibitory potency of AMPs against all pathogenic bacteria, and tsAMP-CS predicts precise MIC values of the AMP sequence against multiple strains of 10 pathogenic species.

The tsAMP framework introduces several methodological improvements over conventional data processing methods (Figure 1). In tsAMP-I module, positive samples consist of 19,412 unique AMP sequences curated from dbAMP2.0 (Jhong et al., 2022) and DBAASP (Pirtskhalava et al., 2021) databases to enhance model generalizability, while negative samples were drawn from non-AMP sequences in UniProt (UniProt Consortium, 2019) and environmentally sourced sequences from the Global Microbial Gene Catalog (GMGC) (Coelho et al., 2022) database. To ensure comparability, a subset of negative samples was truncated to match the length distribution of positive samples. Following initial feature extraction using ESM-1v, physicochemical properties specific to AMPs were integrated into the feature embedding vectors. The model robustness was further improved through introducing controlled noise into the representations of positive samples.

The tsAMP-C module operates as a binary classification model; it distinguishes whether the MIC of a given AMP against a specific target species lies above or below a predefined threshold. Samples with MIC values above the threshold were assigned class 1 (which indicates weak inhibitory activity of the AMP against bacterial species), and those below the threshold were assigned to class 0 (which denotes a strong inhibitory activity of the AMP against the bacterial species). To address variability of MIC distributions across different target species, we selected thresholds to ensure a relatively balanced class distribution. A generative adversarial network (GAN) was used before model training to augment the concatenated sequence feature representations of AMP and target species.

For the tsAMP-CS module, exact MIC values were predicted for 10 target species’ strains, with more than 1,000 samples, after standardization to a logarithmic scale (log μM) (Sharma et al., 2023).

### 3.2 Comparative prediction of AMP identification performance

**Figure 2:**
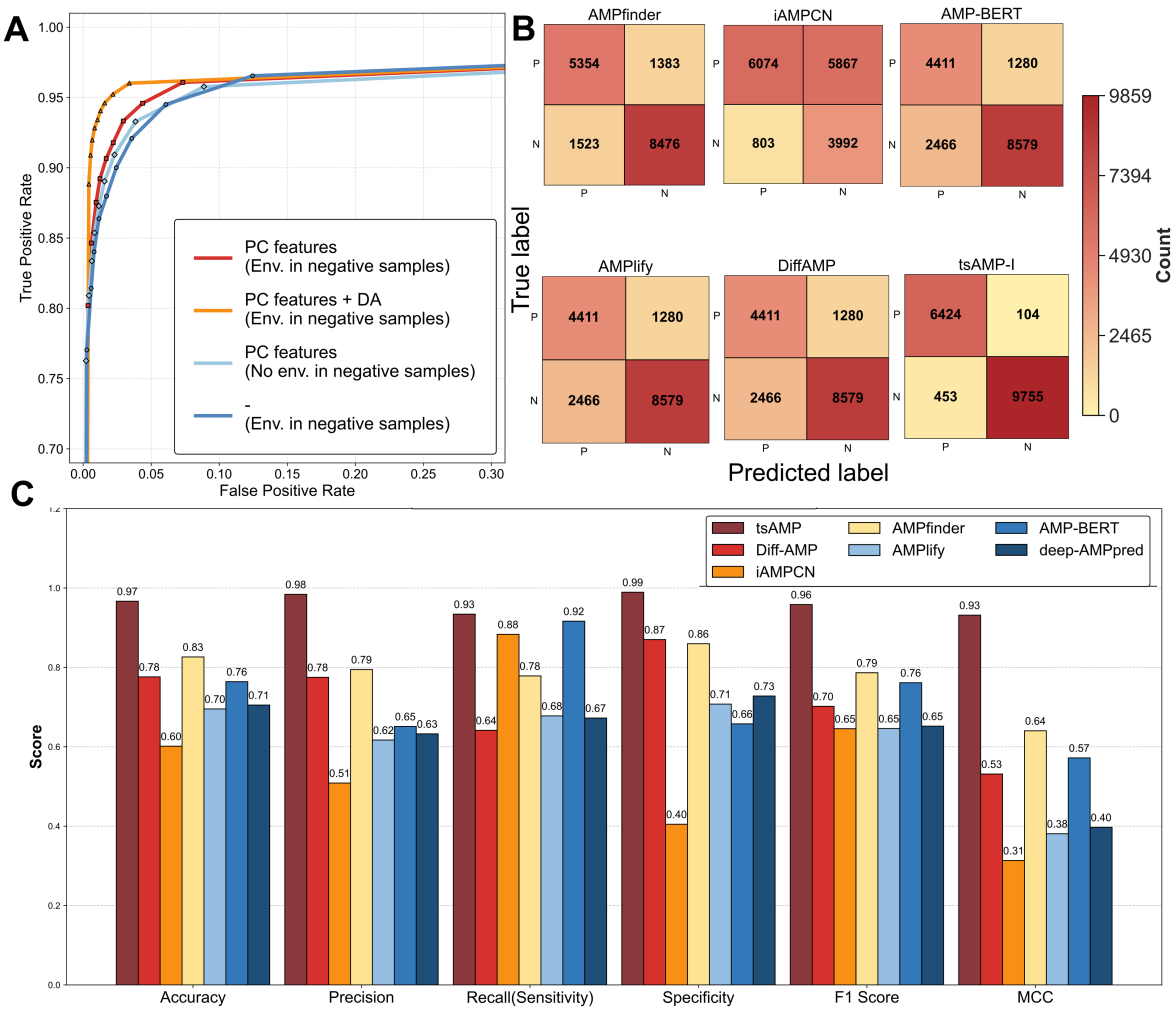
Performance of tsAMP-I. **(A)** ROC curves from ablation studies assessing different model configurations: inclusion/exclusion of physicochemical properties, data augmentation implementation, and completeness of negative samples. **(B)** Confusion matrices comparing model performance. tsAMP-I demonstrates a superior predictive accuracy for positive samples, with 453 negative samples misclassified as false positives. **(C)** Comparative performance benchmarking against existing AMP prediction tools.

Three optimization strategies were employed through systematic ablation experiments: (1) in addition to using ESM-1v to extract AMP sequence features, physicochemical properties were incorporated into the features; (2) training samples were augmented with noise perturbations; and (3) negative samples were expanded by including environmental negative sequences. The receiver operating characteristic (ROC) curves in Figure 2A show the corresponding ablation results. The model configuration combining physicochemical properties, data augmentation, and complete negative-sample configuration yielded crucial performance gains. Specifically, the model with physicochemical properties outperformed others in terms of the low FPR interval (<0.3) and achieved a specificity of 98.42%, notably higher than the 96.43% obtained in the model without physicochemical properties. Furthermore, the model that combined physicochemical properties with data augmentation exhibited the best performance, with a TPR of 92.83% at FPR = 0.01. In contrast, other models require relaxation to FPR > 0.02 to reach comparable TPR levels. The inclusion of environmental sequences in the negative dataset further improved specificity by 3.5%, especially at FPR = 0.02, where the model with environmental samples achieved 97.81% specificity compared with 94.17% without them.

Further, tsAMP-I was compared against six state-of-the-art AMP prediction models: Diff-AMP, iAMPCN, AMPfinder, AMPlify, AMP-BERT and deep-AMPpred (Jhong et al., 2022; Lee et al., 2023; Li et al., 2022; Wang et al., 2024; Xu et al., 2023; Zhao et al., 2025). Figure 2B shows the confusion matrices of the six models predicted on the same test set. tsAMP-I achieved markedly fewer false positives (104), than all benchmarks. Performance metrics of all seven models are summarized in Figure 2C. The specificity of tsAMP-I reached (0.989 ± 0.0011), which is 52.9% higher than iAMPCN. In terms of the overall performance, tsAMP-I attained an F1 score of 0.958 ± 0.0026, exceeding that of the suboptimal model, AMPfinder (0.79). This finding indicates that tsAMP-I maintained a high recall rate of 0.934 (95% CI: [0.9319, 0.9374]), while substantially reducing false positives, as reflected by its high accuracy (0.984) (95% CI: [0.9791, 0.9847]). In addition, the Matthews correlation coefficient (MCC) reached (0.932 ± 0.0029), close to the theoretical maximum value of 1, indicating strong agreement between predictions and ground truth. This value substantially outperformed those of other models (MCC < 0.65), underscoring the robustness of the tsAMP-I in handling class-imbalanced datasets.

Specifically, a rigorous test set was designed to predict the model’s capability in identifying non-homologous AMPs and resisting bias from trivial physicochemical features. Positive sequences were clustered using CD-HIT, and only those sharing <40% identity with the training set were retained (Supplementary Figure S1A). Furthermore, a “hard negative” test set was constructed by selecting non-AMP peptides with physicochemical properties (net charge and isoelectric point) that significantly overlap with true AMPs (Supplementary Figure S1B). Despite these stringent constraints, high performance was maintained by the model, with a precision of 0.9431, recall of 0.8669, and F1-score of 0.9029 being achieved.

### 3.3 Prediction of the antibacterial capability of AMPs against different pathogens by tsAMP-C

**Figure 3:**
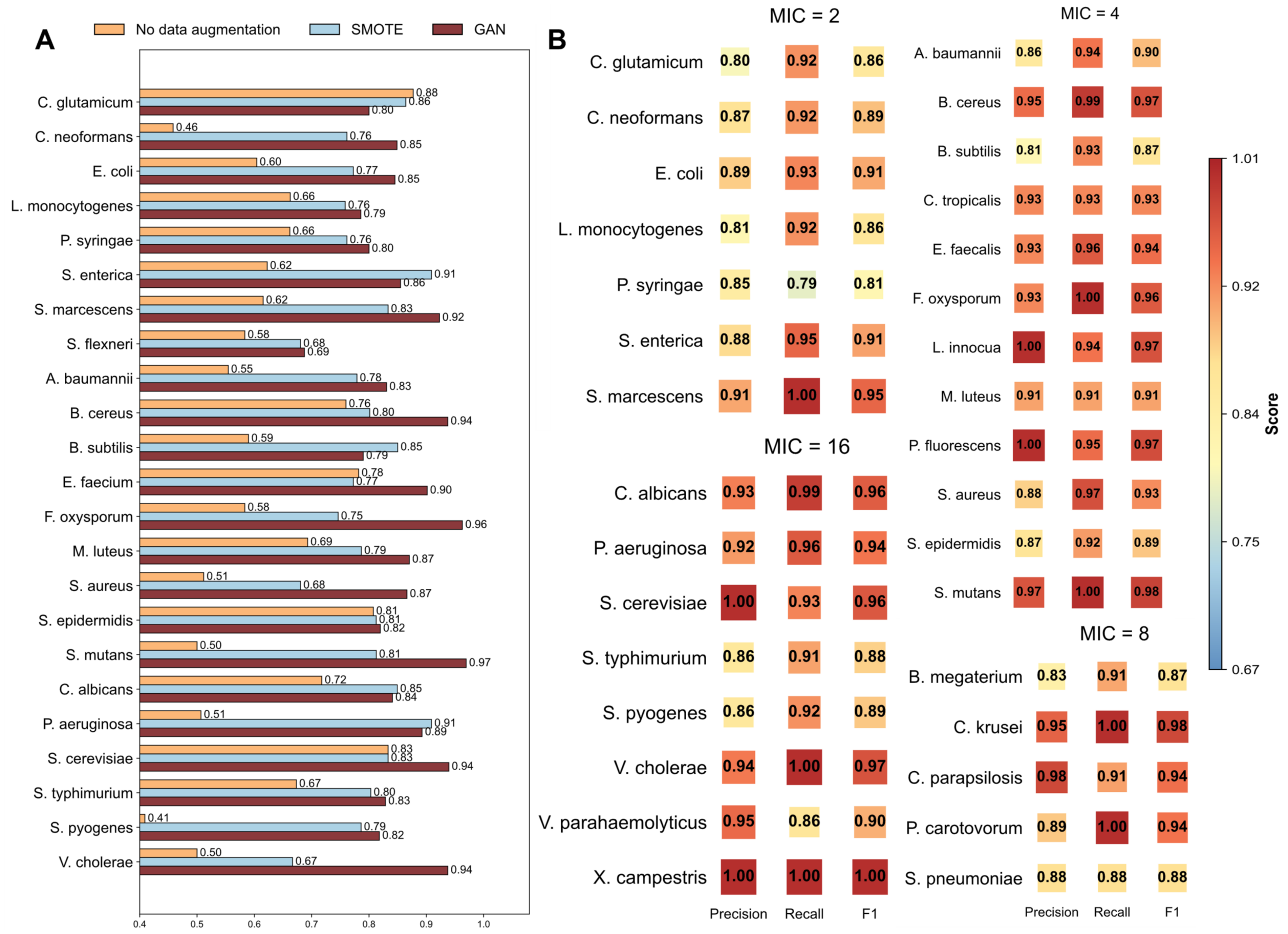
Performance of tsAMP-C. **(A)** Effect of SMOTE and GAN-based data augmentation on MIC range prediction. Data augmentation improved prediction capability across all target species. **(B)** Performance metrics (precision, recall, and F1-score) of tsAMP-C for MIC range classification across 33 target species, where samples below MIC thresholds were labeled as positive and those above as negative.

Antimicrobial potency was classified for a preliminary assessment of AMP inhibitory efficacy. To predict MIC values across various target species, we initially selected 33 target species from the DBAASP and DBAMP 2.0 databases based on their AMP targeting frequency (>100 AMPs). The corresponding MIC range was determined for each target species. GAN and SMOTE were then employed to generate synthetic AMP-like sequences to enhance the training dataset (Chawla et al., 2002; Goodfellow et al., 2020). Data augmentation consistently improved MIC prediction accuracy across all tested species (Figure 3A), with the most significant improvement observed in Gram-negative bacteria. Particularly, the prediction accuracy for *Pseudomonas aeruginosa* increased dramatically from 0.51 to 0.83 after augmentation. Overall, the augmented model achieved >85% accuracy for most target species.

Figure 3B further demonstrates the performance of tsAMP-C in predicting minimum MIC ranges across 33 target species. Notably, tsAMP-C exhibited an excellent predictive capability for clinically highly prevalent pathogens, such as *Escherichia coli* (F1 = 0.801 ± 0.0092), *Staphylococcus aureus* (F1 = 0.763 ± 0.0255), and *Pseudomonas aeruginosa* (F1 = 0.774 ± 0.0362). These high-performing pathogens were subsequently selected for the refined MIC value prediction.

Beyond predicting the inhibitory potency of various AMPs against 33 target pathogens, we developed a more generalized model that demonstrates robust predictive capability across 156 previously unseen pathogen species. When an inhibition threshold of 8 μM was used for classification, the model achieved balanced performance with a precision of 0.7465 (95% CI: [0.7453, 0.7492]), a recall of 0.7290 (95% CI: [0.7222, 0.7297]), and an F1-score of 0.7376 (95% CI: [0.7358, 0.7399]). These results indicate reliable generalization and consistent predictive stability across evolutionarily diverse pathogenic taxa.

### 3.4 Strain-level AMP MIC prediction capability of tsAMP-CS targeting pathogens

**Figure 4:**
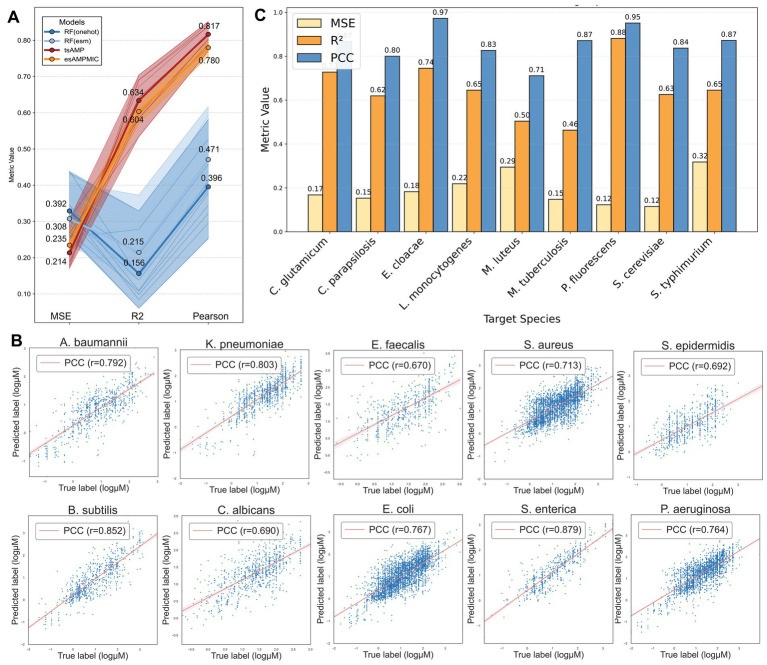
Performance of tsAMP-CS. **(A)** Comparative analysis of tsAMP-CS against one-hot-encoded decision trees, ESM-encoded decision trees, and the deep learning model esAMPMIC. **(B)** Fivefold cross-validation results showing the test set PCC values consistently above 0.77, with *Acinetobacter baumannii* reaching 0.852. **(C)** Prediction of MIC values for strains of target species not encountered during training. Strains belonging to the same species were grouped together for performance prediction.

Classifying the inhibitory potency of AMPs provides a preliminary prediction of their antibacterial effects. However, accurate determination of the specific MIC values of AMPs against different pathogenic bacteria requires comprehensive consideration of strain-specific characteristics. To further predict the capability of AMPs to predict precise MIC values against target pathogenic bacteria species, we developed an MLP-based regression model (tsAMP-CS) and systematically compared its performance against two baseline approaches: a random forest (RF) model using ESM-1v feature extraction and another RF model using one-hot encoding. Our architecture combined with ESM-1v embeddings outperformed both baselines in modeling AMP-target species interactions (Figure 4A). For MIC value prediction across 10 distinct target species, tsAMP-CS achieved an average MSE of 0.214 (95% CI: [0.2132, 0.2208]), significantly lower than that of the RF models (MSE > 0.3). Concurrently, it attained a higher coefficient of determination (*R*^2^ = 0.634 ± 0.0442) and Pearson correlation coefficient (PCC, *r* = 0.817 ± 0.0264), which indicate its strong predictive reliability. In contrast, the two RF models (especially the one-hot coded version) displayed the weaker performance, with the largest prediction errors observed for *Bacillus subtilis* (MSE > 0.311). Moreover, tsAMP-CS outperformed the current state-of-the-art model, esAMPMIC (Figure 4A). To quantify the contribution of each augmentation strategy, we conducted an ablation study on *Escherichia coli* strain-level MIC prediction data (Supplementary Table S1). The baseline model without augmentation achieved a Val *R*^2^ of 0.449. Each strategy independently improved performance, with dropout noise and random scaling contributing the most. The full combination of all four strategies yielded the best results (Val *R*^2^ = 0.733), confirming their complementary benefits.

As presented in Figure 4B, the scatter plot of the predicted versus true MIC values showed a distinct diagonal distribution pattern (Pearson’s *r* = 0.81 ± 0.04), which reflects the reduced absolute error and accurate capture of the linear relationship underlying MIC variation across species. tsAMP-CS also maintained a robust MIC prediction capability even for AMP-target strain pairs with limited training data. The model successfully predicted MIC values of 279 bacterial strains in response to AMPs, with 156 strains achieving an *R*^2^ > 0.5 during the test. Strong performance was observed across 15 strains of *Escherichia coli* and *Staphylococcus aureus* (Table 1; Supplementary Table S2). For high-sample strains (e.g., *Staphylococcus aureus* USA, *Escherichia coli* ATCC 8739) and low-sample strains (e.g., *Staphylococcus aureus* K52 and *Escherichia coli* DSM 787), the model consistently achieved *R*^2^ values above 0.6. Notably, for *Staphylococcus aureus* Newman, which has a wide MIC range, tsAMP-CS maintained a high PCC (0.914) despite having a high MSE (0.765), which further underscores its robust predictive power for MIC values.

**Table 1:** Performance prediction of tsAMP-CS on strains of *Staphylococcus aureus* and *Escherichia coli* (*n* ≥ 10).

Bacterial strain	Count	MSE	R2	Pearson
*Staphylococcus aureus* 725	14	0.039	0.613	0.832
*Staphylococcus aureus* ATCC 1717	14	0.031	0.850	0.986
*Staphylococcus aureus* ATCC 33592	55	0.232	0.626	0.863
*Staphylococcus aureus* ATCC 700699	31	0.048	0.902	0.959
*Staphylococcus aureus* DSM 1104	31	0.130	0.720	0.862
*Staphylococcus aureus* DSM 6247	43	0.058	0.655	0.812
*Staphylococcus aureus* Newman	42	0.765	0.672	0.914
*Staphylococcus aureus* SA113	25	0.278	0.597	0.886
*Staphylococcus aureus* USA 300	185	0.129	0.727	0.860
*Escherichia coli* AB94012	11	0.028	0.939	0.971
*Escherichia coli* ATCC 25726	61	0.088	0.743	0.866
*Escherichia coli* ATCC 8739	182	0.258	0.708	0.851
*Escherichia coli* ATCC 9637	10	0.019	0.869	0.937
*Escherichia coli* DSM 1116	32	0.115	0.723	0.852
*Escherichia coli* HB101	25	0.127	0.741	0.879
*Escherichia coli* MC4100	49	0.027	0.817	0.915
*Escherichia coli* MG1655	96	0.123	0.715	0.869
*Escherichia coli* ML-35p	99	0.161	0.816	0.927
*Escherichia coli* W3110	113	0.133	0.690	0.837

Count indicates the number of isolates of each strain included in the test set. Strains with *n* < 10 are reported separately in Supplementary Table S1.

Similar to tsAMP-C, we implemented tsAMP-CS with an enhanced generalizability to predict the MIC values of AMPs against novel strains of previously untargeted species. The unified model attained satisfactory performance on previously unencountered strains (*R*^2^ = 0.5090 ± 0.0308, MSE = 0.3918 ± 0.0158, and PCC = 0.7174 ± 0.0183). As demonstrated in Figure 4C, tsAMP-CS exhibited strong predictive capability across nine newly targeted species, with outstanding performance for *Pseudomonas fluorescens* (MSE = 0.123, *R*^2^ = 0.881, and PCC = 0.951) and *Enterobacter cloacae* (MSE = 0.182, *R*^2^ = 0.744; PCC = 0.972). This outcome confirms its robust strain-level MIC prediction accuracy across diverse pathogens.

### 3.5 External computational benchmarking of MIC predictions

To assess the computational generalizability of tsAMP, we benchmarked its predictions against two independent sets of previously published experimental MIC data.

Based on MIC values reported in the literature for experiments involving new AMPs and various bacterial strains, the MIC values of 10 AMPs against the target strain *Micrococcus luteus* were predicted using tsAMP (Goldberg et al., 2025) (Supplementary Table S3). In the inhibition potency assessment conducted with tsAMP-C, a threshold of 16 μg/mL was selected, resulting in a prediction accuracy of 80%, with AMPs exhibiting weak inhibitory activity being identified with 100% accuracy. When tsAMP-CS was applied to predict the MIC for *Micrococcus luteus*, log_10_ transformation results showed an MSE of 0.1489. The true and predicted MIC values of these 10 AMPs were ranked, and ranking metrics including NDCG (0.791), MAP (0.698) were employed to screen for the most highly promising AMPs. The model’s predictions were used as a ranking signal and demonstrated strong consistency with the ground-truth ranking, particularly regarding overall list quality, as reflected by NDCG and MAP.

To evaluate the predictive performance of tsAMP-CS on independently generated AMPs, we collected MIC data for 10 novel AMPs produced by ProteoGPT (Wang et al., 2025). These peptides were experimentally tested against three representative pathogen strains spanning both prokaryotic and eukaryotic kingdoms: *Escherichia coli* ATCC 25922 (Gram-negative), *Staphylococcus aureus* ATCC 25923 (Gram-positive), and *Candida albicans* ATCC 10231 (fungal pathogen). Across all three targets, log₁₀-transformed predictions demonstrated strong correlation with experimental values, with Pearson correlation coefficients of 0.87, 0.75, and 0.66 for *E. coli*, *S. aureus*, and *C. albicans*, respectively. Notably, the model achieved consistently high-ranking performance across all three strains, with C-index values exceeding 0.82 and NDCG@10 scores above 0.96 in each case, indicating reliable prioritization of potent AMP candidates regardless of the target organism. For *E. coli*, the model attained the lowest log₁₀ MSE (0.038), while correctly identifying all three least active AMPs (Bottom-3 Hit Ratio = 100%). For *S. aureus* and *C. albicans*, the Top-5 and Bottom-5 Hit Ratios both reached 80%, demonstrating balanced discrimination at both ends of the activity spectrum. These results indicate that tsAMP-CS generalizes effectively to novel designed peptides and maintains robust ranking capacity across phylogenetically diverse targets.

We also benchmarked our predictions against an independent dataset of 51 published experimentally determined relative MIC values of four antimicrobial peptides (PROA, PGLA, IND, and PYR) against various *E. coli* strains. The relative MIC was defined as the ratio of the MIC determined for a specific resistant strain to the MIC determined for the standard *E. coli* K-12 reference strain for each respective peptide (Lázár et al., 2018). As illustrated in the Supplementary Table S4, the model successfully characterized the relative potency shifts across diverse biological backgrounds, achieving high ranking accuracy for peptides with extensive strain sets, such as PROA (*N* = 17) and IND (*N* = 13), which yielded NDCG scores exceeding 0.85 and positive Spearman correlations (0.50 and 0.32, respectively). Furthermore, the model exhibited a balanced ability to identify both high-potency and low-sensitivity combinations, particularly evidenced by the Bottom-5 Hit Ratios of 0.80 for both PROA and IND, which is consistent with the model’s ability to computationally rank strain-level susceptibility differences, suggesting that the global biochemical context captured by the mean proteome embedding correlates with strain-level differences in predicted inhibitory activity.

### 3.6 MIC prediction for the Mgnify_genome database and putative AMP candidate identification using tsAMP

The Mgnify_genome database (Almeida et al., 2021) refers to a human gut microbial proteomic resource covering 70% uncultured species and 40% functionally unannotated proteins; containing over 170 million protein sequences derived from 4,644 gut prokaryotic species. Notably, the database provides a pre-computed protein catalog^1^ in which protein sequences have already been predicted from metagenomic assemblies and clustered at 50% sequence identity by the MGnify consortium, allowing direct use without additional nucleotide-to-protein translation. The tsAMP-I model was employed to screen 962,603 sequences (length < 50 AA) from this database, and it identified 8,277 putative AMPs. Among these AMPs, 5,025 sequences (60.7%) exhibited a net charge between +1 and +9, and 6,168 sequences (74.5%) displayed a notable amphipathicity. Subsequent MIC predictions for these candidate AMPs, along with known AMPs from DBAMP and DBAASP, were performed using tsAMP-C and tsAMP-CS.

**Figure 5:**
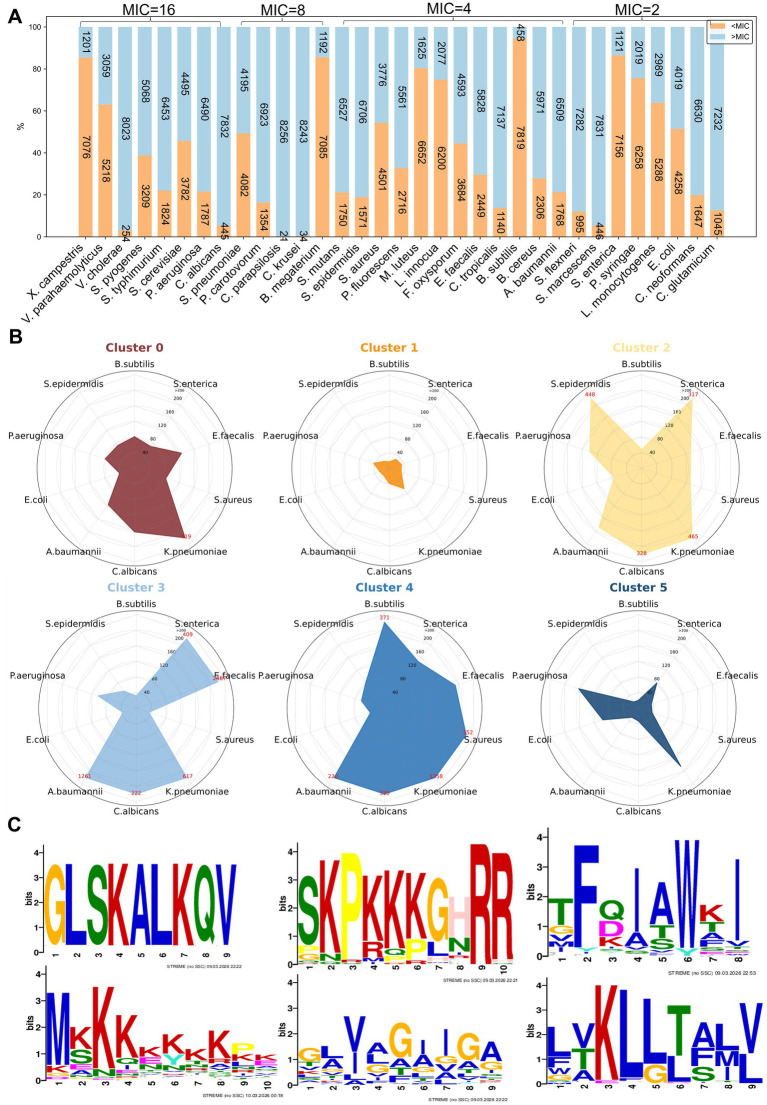
Statistics of novel AMP discoveries. **(A)** Distribution of MIC value ranges predicted by tsAMP-C for novel AMPs. **(B)** Results of AMP clustering. The AMPs were clustered through the concatenation of their MICs against 10 targeted species into 10-dimensional vectors. **(C)** Representative motifs of each cluster.

Preliminary MIC ranges for AMP interactions with 33 target species revealed significantly higher AMP susceptibility in *Bacillus subtilis* (Figure 5A). Detailed MIC prediction for 10 target species (Supplementary Figure S3A) further confirmed that *Bacillus subtilis* exhibited the highest frequency of AMP interactions at identical MIC values. Notably, at an MIC threshold of 15 μM, over 5,070 AMPs showed broad-spectrum activity against two or more target species (Supplementary Figure S2B).

Several strains of *Escherichia coli* were selected to predict strain-specific MIC variations. Using tsAMP-CS, the same AMP exhibited differential MIC distributions across strains (Supplementary Figure S2C). Among the six AMPs tested, two showed higher predicted MICs against *Escherichia coli* LF82 than against UTI89, while the opposite trend was observed for the remaining four AMPs, which confirms that strain specificity considerably influences AMP MIC values.

To investigate patterns in antibacterial activity, a 10-dimensional feature vector was constructed for each AMP based on its average MIC values against 10 target bacterial strains. Clustering analysis of all 8,277 feature vectors was performed, with performance predicted using the Silhouette coefficient, which measures intra-cluster compactness and inter-cluster separation. As displayed in Figure 5B and Supplementary Figure S3D, the Silhouette coefficient peaked at a cluster number (k) of 6, which indicates that six clusters best reflected the differential patterns among the AMPs. A two-dimensional visualization of the clustering results after principal component analysis (PCA) is presented in Supplementary Figure S2E. For Clusters 0, 1, and 5, the representative motifs GLSKALKQV, SKPKKKGHRR, and LVKLLTALV were found to be cationic and enriched with amphipathic and hydrophobic residues. For the highly aggregated Clusters 2, 3, and 4, representative sequences such as TFQIAWTI, MKKKKKKKPK, and GLVAGIIGA were identified, which sequences were characterized by a positive charge or high hydrophobicity (Figure 5C).

## 4 Discussion

The tsAMP-I module incorporates three key improvements. First, the negative-sample construction strategy was substantially expanded and refined. Environmental sequences from AMP distribution habitats were incorporated to enhance model generalization, which suppressed false positives and boosted recall from 0.880 to 0.934. However, negative-sample construction remains a widely recognized challenge in AMP prediction, as experimentally confirmed non-AMP sequences are virtually nonexistent in current databases (Sidorczuk et al., 2022). Most existing tools rely solely on keyword-based filtering of UniProt, which cannot guarantee the absence of unannotated antimicrobial peptides. To address this, we implemented a multi-layered mitigation strategy: (i) BLAST analysis at a 40% sequence identity threshold was applied to remove sequences similar to known AMPs; (ii) negative-sample sources were diversified by incorporating both UniProt reviewed proteins and GMGC environmental sequences, reducing the risk of over-representing any particular protein family; and (iii) the effectiveness of this strategy was independently assessed using external datasets from TG-CDDPM and AmpHGT (Cao et al., 2025; He et al., 2025), yielding a prediction accuracy of 94.58%. A small fraction of unannotated active peptides may still reside in the negative set, which remains a limitation shared by all current AMP predictors. Second, physicochemical properties were incorporated during feature extraction, further enhancing AMP identification. Third, controlled noise perturbations were strategically integrated during training to strengthen the model’s robustness and generalization. Prediction MAE scales predictably with noise intensity and remains below 0.09 even when noise is quadrupled to σ = 0.20 (Supplementary Figure S1C), which we regard as an approximate critical stability threshold beyond which extreme noise (e.g., σ = 0.40) begins to obscure intrinsic feature patterns and degrade predictive performance.

A notable feature of the tsAMP-C module is its capability to predict inhibitory concentrations between completely novel AMPs and previously unencountered pathogens. tsAMP-C employs GAN-perturbed ESM feature vectors to explore the local functional manifold around known peptides, and it synthesizes biologically plausible embeddings that encapsulate the evolutionary and structural semantics of training data to augment the dataset and mitigate the impact of experimental noise and inter-lab heterogeneity. Systematic ablation studies confirmed the superiority of this GAN-based strategy over traditional methods; while SMOTE-based linear interpolation often failed to capture the complex, non-linear constraints of the ESM-1v latent space, GAN-guided augmentation provided a significant boost in accuracy. In Supplementary Figure S1D, high-dimensional visualization (t-SNE) further supports this approach, showing that synthetic embeddings do not merely diffuse into the background noise but precisely populate the local neighborhoods of real feature clusters, thereby maintaining high generative fidelity. The impact of this augmentation is most pronounced when stratified by sample size (Supplementary Figure S1E): for rare strains with limited experimental records, the GAN-informed latent exploration effectively compensates for data sparsity, allowing the model to learn robust decision boundaries where empirical data is otherwise insufficient (Bhattarai et al., 2025; Zervou et al., 2025).

In the tsAMP-C module, inhibitory potency against 33 species was predicted using optimized MIC thresholds (2, 4, 8, 16 μM) that reflect biological susceptibility variations. This distribution-based selection prevents class imbalance and enhances discriminative performance by identifying candidates exceptionally potent relative to a specific pathogen’s intrinsic baseline. To validate generalization, we mapped the predictive performance of 105 unseen species onto a phylogenetic tree. As illustrated in Supplementary Figure S3, robust performance (F1-score: 0.7376) was consistently maintained across diverse clades, even for taxa at significant evolutionary distances from the training set. The sporadic distribution of outliers indicates that the model’s predictions are consistent with the presence of shared sequence-function patterns across diverse taxa, though whether these reflect universal antimicrobial motifs requires further experimental investigation.

Different strains of the same bacterial species, despite sharing highly conserved core genomes, may exhibit genetic and phenotypic variations that influence AMP susceptibility. For the extraction of strain-level features, the mean proteome embedding calculated from deduplicated unique protein sequences was selected. This approach was found to consistently outperform or match the performance of specialized subsets, such as resistance gene or membrane protein embeddings in Supplementary Figure S1F. While targeted subsets (resistance genes, membrane proteins) are functionally relevant, they represent only a fraction of the cellular machinery involved in AMP response. Comparative proteomic studies have shown that AMP susceptibility differences between strains are associated with proteins distributed across diverse functional categories, including outer membrane proteins, efflux pump components, cell wall biosynthesis enzymes, chaperones, and oxidative stress-response proteins, rather than being confined to a single pathway. Meanwhile, strain-specific accessory proteins such as plasmid-encoded transporters and horizontally acquired modification enzymes further modulate susceptibility (Cardoso et al., 2017; Kintses et al., 2019; Rosconi et al., 2022). Since ESM-1v embeddings encode both sequence and structural-functional properties of proteins, the mean proteome embedding implicitly reflects the global distribution of these diverse protein categories across a given strain. By contrast, restricting embeddings to resistance genes or membrane proteins alone discards information from regulatory, metabolic, and accessory proteins that collectively shape the AMP response at the whole-cell level. These results suggest that strain-level MIC variation is driven by the coordinated action of the entire proteome rather than isolated protein categories (Koblitz et al., 2025; Sethi et al., 2026).

To enhance predictive capability for strains with limited sample sizes, the tsAMP-CS module integrates a comprehensive augmentation strategy (Gaussian noise, dropout noise, random scaling, and mixup interpolation). Even for strains with fewer than 50 samples, such as *Staphylococcus aureus* ATCC 700699 and *Candida albicans* ATCC 37092, tsAMP-CS achieved high performance (*R*^2^ > 0.8, MSE < 0.2). Ultimately, the model identified 156 AMPs with strain-level MIC estimations across diverse bacterial strains. Beyond absolute MIC values, the tsAMP models function as reliable *in silico* “intelligent sorters,” enhancing the efficiency of experimental validation (NDCG = 0.791, MAP = 0.698).

The tsAMP model identified 8,277 putative AMP candidates, which should be noted that these candidates were predicted computationally. To assess the statistical reliability of the 8,277 computationally predicted AMP candidates, we implemented a computational validation framework. A sequence shuffling control experiment was conducted to disrupt biological patterns; the inability to recover any significant motifs or consistent clusters from the shuffled data effectively eliminates the risk of a high false discovery rate. This is further substantiated by the exceptionally low E-values (3.8e-093 to 4.6e-008) of the identified motifs, indicating they are highly non-random and evolutionarily conserved. To further characterize the putative antimicrobial mechanisms, we classified all 8,277 candidates based on their physicochemical properties including net charge, hydrophobic moment, and Boman index (Aguilera-Puga and Plisson, 2024; Santos-Júnior et al., 2024; Wang et al., 2023). These classifications should be regarded as hypothetical assignments rather than experimentally confirmed mechanisms of action. The analysis revealed a diverse mechanistic landscape dominated by electrostatic disruption (26.4%) and cell-wall penetration (21.6%), with additional candidates consistent with carpet model (9.6%), hydrophobic insertion (10.0%), and toroidal pore (3.2%) mechanisms (Supplementary Figure S4) (Wimley, 2010). Future work incorporating molecular dynamics simulations would provide atomistic-level validation of these predicted mechanisms (Tian et al., 2025; Wang, 2026). The strong alignment between these motifs and essential antimicrobial hallmarks suggests that the predicted candidates share conserved physicochemical characteristics consistent with known antimicrobial features rather than representing stochastic artifacts, providing computational evidence consistent with potential antimicrobial function, which warrants future experimental verification. While tsAMP identified 8,277 putative AMP candidates with predicted strain-level MIC values, further prioritization is needed to guide experimental validation. In practice, candidates could be filtered through toxicity and hemolytic activity prediction (e.g., ToxinPred, HemoPI), novelty assessment against existing AMP databases such as APD3 and DRAMP, structural diversity analysis via physicochemical clustering to maximize chemical space coverage, and synthesis feasibility screening based on peptide length and structural complexity. Integrating these criteria with tsAMP’s MIC predictions would enable researchers to narrow the candidate set to an experimentally tractable subset.

Despite the robust predictive capabilities of the tsAMP modules, several limitations must be acknowledged. First, because MIC training data were aggregated from multiple databases with varying assay protocols, media compositions, inoculum densities, and endpoint determination methods, inter-laboratory variability may introduce systematic noise that partially accounts for residual prediction error, a limitation shared by all current data-driven AMP-MIC prediction studies, as highlighted by APD6 guidelines (Wang et al., 2026). Although we applied CD-HIT clustering at 40% sequence identity and verified train-test separation via BLASTP (Supplementary Figure S2A), residual biases from overrepresented peptide families in public AMP databases cannot be entirely excluded. As discussed above, residual label noise from potentially unannotated AMPs in the negative dataset cannot be entirely excluded, although our multi-layered filtering strategy and external validation results suggest that its impact on model performance is modest. Second, due to extreme data skewness in early-stage AMP candidate identification, it remains challenging to apply established clinical breakpoints (e.g., CLSI/EUCAST); therefore, the distribution-based MIC thresholds (2–16 μM) used in this study reflect relative biological susceptibility rather than absolute clinical standards. Third, while our ablation experiments confirmed that mean proteome embeddings outperformed resistance gene or membrane protein subsets alone (Supplementary Figure S1F), averaging across the entire proteome inevitably introduces noise and may obscure the contribution of specific resistance determinants. Future work could incorporate attention-based mechanisms to weight individual proteins by their functional relevance. Additionally, as tsAMP relies on ESM-1v for sequence encoding, the current framework is most applicable to canonical L-amino acid linear peptides; its predictive utility for D-amino acid peptides, cyclic peptides, or non-ribosomal peptides remains limited and has not been evaluated. Furthermore, it should be noted that this study is entirely computational. The 8,277 putative AMP candidates have not been experimentally validated through peptide synthesis, MIC assays, or toxicity testing, and should be interpreted as computational estimates to guide experimental prioritization.

## 5 Conclusion

The tsAMP framework, consisting of three specialized functional modules, was developed based on the ESM-1v protein language model. The first module (tsAMP-I) performs binary classification to distinguish AMPs from non-AMPs. The second module (tsAMP-C) estimates MIC ranges against 33 target species, while the third module (tsAMP-CS) provides strain-level MIC estimations across 279 strains from 10 prevalent microbial targets. Using this framework, 8,277 putative AMP candidates were identified, and their MIC value distributions against target bacterial strains were computationally characterized. Overall, these findings underscore the utility of tsAMP for large-scale AMP candidate identification and strain-level MIC estimation, offering a computational tool to support antimicrobial peptide research and assist future experimental investigations.

## Data Availability

The complete source code, benchmark datasets, and pretrained model weights for tsAMP are publicly available on GitHub at https://github.com/YangLab-BUPT/tsAMP.

## Footnotes

1
https://ftp.ebi.ac.uk/pub/databases/metagenomics/mgnify_genomes/human-gut/v2.0.2/protein_catalogue/
