# Supplementary material for: tsAMP: a strain-level antimicrobial peptide identification framework based on large language models and pathogen genomic variation
Source: Front Microbiol. 2026 Jul 2;17:1842380. doi: 10.3389/fmicb.2026.1842380 (PMC13372748; doi:10.3389/fmicb.2026.1842380)
Supplement: Supplementary file 1 [file Data_Sheet_1.DOCX]

Supplementary Material

# Supplementary Figures and Tables.

##
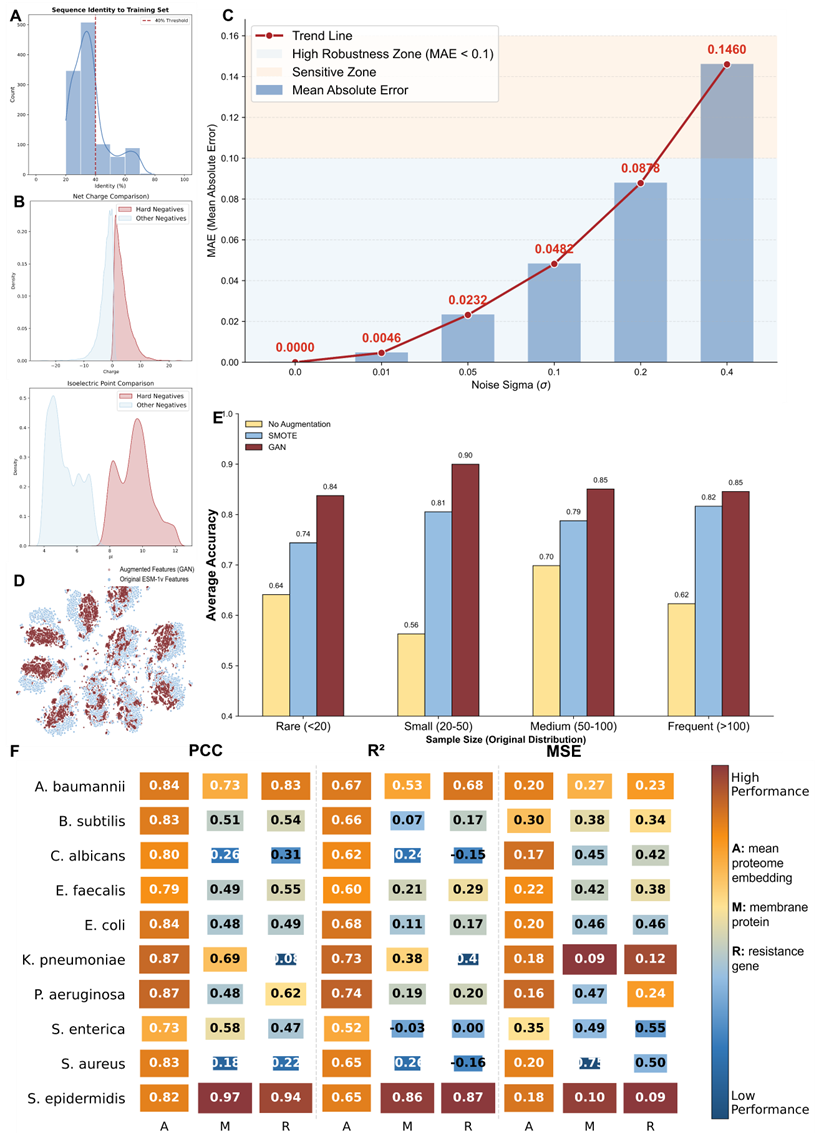
Supplementary Figures

**Fig. S1 Data processing and augmentation analysis. (A)** Homology-aware partitioning of the AMP test set based on BLAST. **(B)** Physicochemical property distributions of hard negative samples. **(C)** Impact of Gaussian noise perturbations on model robustness. **(D)** t-SNE visualization of original and GAN-augmented features. **(E)** Prediction accuracies of different data augmentation strategies. **(F)** Performance matrix of MIC regression across multiple species using different feature extraction methods.

**
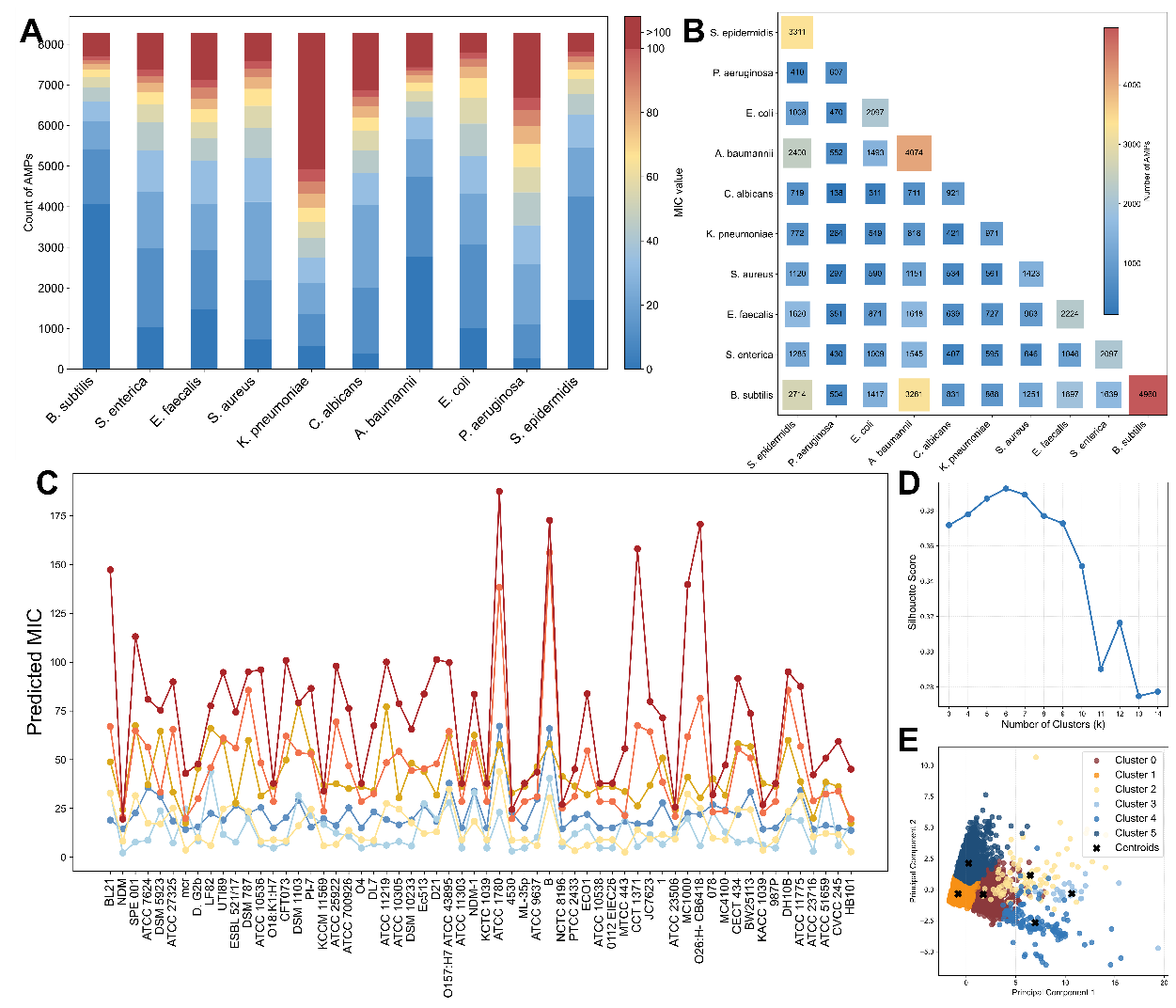
**

**Fig. S2 Statistics of novel AMP discoveries. (A)** Quantitative analysis of AMP-target interactions based on precise MIC values calculated using tsAMP-CS. **(B)** Heatmap displaying AMPs with dual-target activity. At a 15 µM threshold, 3,281 AMPs demonstrated simultaneous activity against both Acinetobacter baumannii and Bacillus subtilis. **(C)** Silhouette coefficient analysis identifying the optimal cluster number (k = 6). **(D)** Results of AMP clustering. The AMPs were clustered through the concatenation of their MICs against 10 targeted species into 10-dimensional vectors**. (E)** Distribution of MIC values for six AMP sequences across multiple Escherichia coli strains.

**
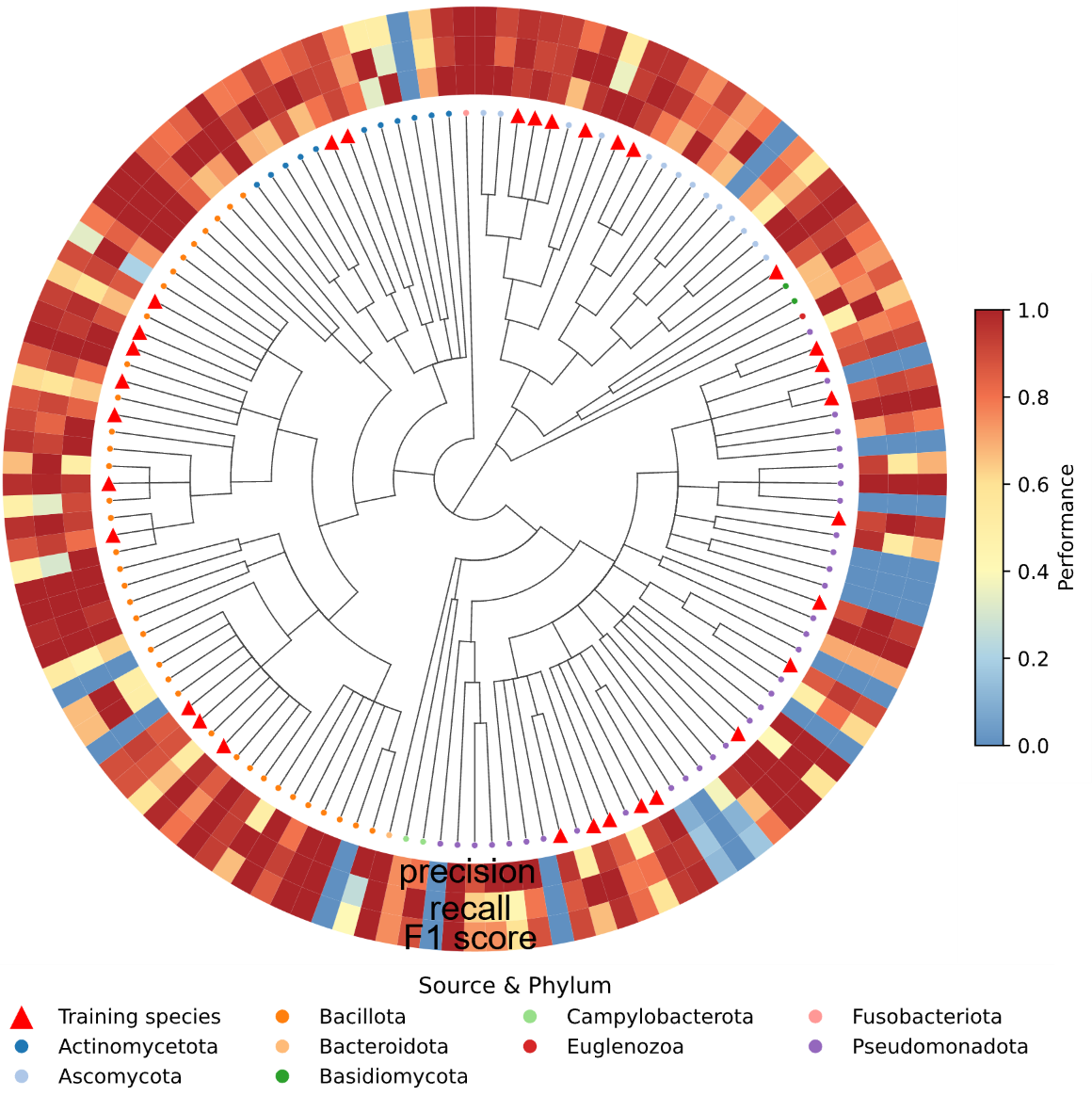
**

**Fig. S3 Evolutionary generalization and predictive performance of tsAMP-C across 105 unseen species.**

**
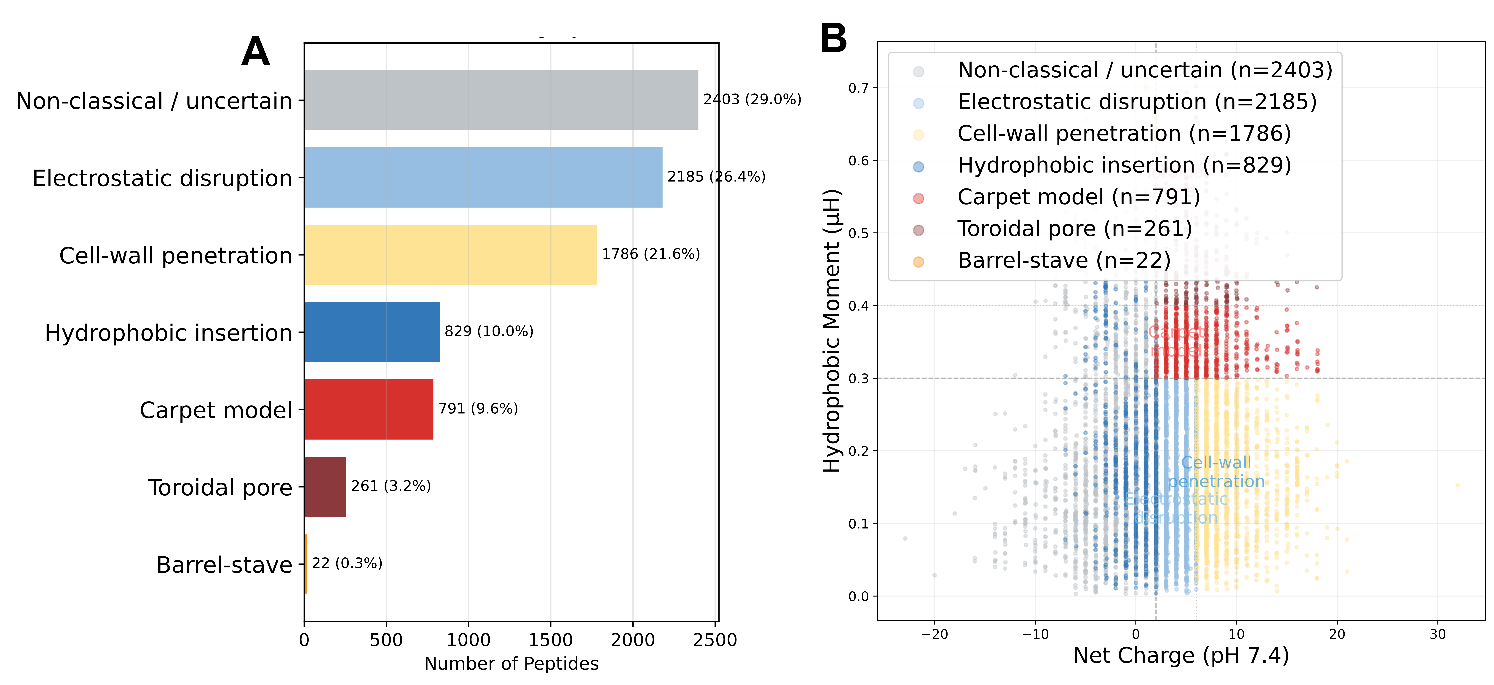
**

**Fig. S4** **Physicochemical mechanism classification of 8,277 putative AMP candidates.** **(A)** Distribution of predicted antimicrobial mechanism categories based on net charge, hydrophobic moment (µH), and Boman index. **(B)** Charge-versus-amphipathicity landscape of all candidates colored by predicted mechanism category.

## Supplementary Tables

| Combination | Val_MSE | Val_R2 | Num_Samples |
| --- | --- | --- | --- |
| All_Augmentation | 0.167 | 0.733 | 31960 |
| All_minus_Gaussian | 0.165 | 0.730 | 22372 |
| All_minus_Dropout | 0.199 | 0.675 | 22372 |
| All_minus_Scaling | 0.211 | 0.655 | 22372 |
| Dropout_Only | 0.238 | 0.622 | 12784 |
| Scaling_Only | 0.246 | 0.609 | 12784 |
| Mixup_Only | 0.257 | 0.590 | 12784 |
| Gaussian_Only | 0.285 | 0.546 | 12784 |
| No_Augmentation | 0.342 | 0.449 | 3196 |

Table S1 ablation result on the MIC regression module ablation result on the MIC regression module.

Table S2 Performance prediction of tsAMP-CS on strains of *Staphylococcus aureus* and Escherichia coli with limited sample sizes (n < 10). These results should be interpreted with caution, as correlation coefficients derived from small samples have wide confidence intervals and limited statistical reliability.

| Bacterial strain | Count | MSE | R2 | Pearson |
| --- | --- | --- | --- | --- |
| Staphylococcus aureus 1056 | 3 | 0.003 | 0.954 | 0.987 |
| Staphylococcus aureus DSM 2569 | 6 | 0.079 | 0.882 | 0.940 |
| Staphylococcus aureus K52 | 4 | 0.088 | 0.743 | 0.964 |
| Staphylococcus aureus NCTC 13277 | 6 | 0.078 | 0.609 | 0.834 |
| Staphylococcus aureus USA 100 | 3 | 0.080 | 0.730 | 0.964 |
| Staphylococcus aureus WKZ2 | 2 | 0.082 | 0.595 | 1.000 |
| Escherichia coli ATCC 10536 | 7 | 0.052 | 0.698 | 0.874 |
| Escherichia coli ATCC 25404 | 5 | 0.071 | 0.901 | 0.991 |
| Escherichia coli DSM 787 | 6 | 0.132 | 0.742 | 0.974 |
| Escherichia coli MC4100 NR698 | 5 | 0.023 | 0.878 | 0.949 |
| Escherichia coli SC9251 | 2 | 0.001 | 0.959 | 1.000 |

**Table S3 Performance prediction of tsAMP on experimental data.**

| AMP | true MIC value | TsAMP-C result | TsAMP-CS result |
| --- | --- | --- | --- |
| GLB1 | 100 | 1 | 16.34332703 |
| RPL41 | 50 | 1 | 33.49515021 |
| CHMP2A | 50 | 1 | 17.08997264 |
| RPS4X | 25 | 1 | 17.14613224 |
| PPP1CB | 25 | 1 | 13.04075045 |
| RPS29 | 12.5 | 0 | 34.82589333 |
| PSMG2 | 12.5 | 1 | 8.151362332 |
| ETS1 | 12.5 | 1 | 34.22575978 |
| DCTN4 | 12.5 | 0 | 21.99369084 |
| DFNA5 | 6.25 | 0 | 4.14153 |

1 indicates that the tsAMP-C predicts a MIC greater than 16 (weak inhibition), while 0 indicates that the tsAMP-C predicts a MIC less than 16 (weak inhibition).

**Table S4 Performance prediction of tsAMP-CS on experimental datasets across various Escherichia coli strains.**

| AMP | Strain Count | Spearman_Rho | NDCG | HitRatio Top5 | HitRatio Bottom5 |
| --- | --- | --- | --- | --- | --- |
| PROA | 17 | 0.497235 | 0.85173 | 0.6 | 0.8 |
| PGLA | 15 | 0.354647 | 0.897164 | 0.4 | 0.4 |
| IND | 13 | 0.321341 | 0.92242 | 0.6 | 0.8 |
| AP | 3 | 0.5 | 0.997953 | 1 | 1 |
| PYR | 3 | 0.866025 | 1 | 1 | 1 |

**
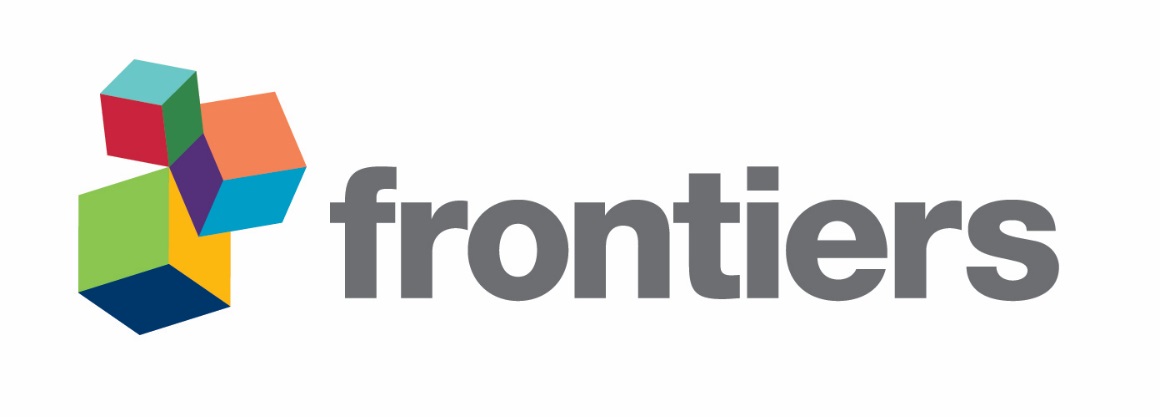
**
